# Public Health Practice Report: water supply and sanitation in Chukotka and Yakutia, Russian Arctic

**DOI:** 10.1080/22423982.2018.1423826

**Published:** 2018-01-31

**Authors:** Alexey A. Dudarev

**Affiliations:** ^a^ Hygiene Department, Northwest Public Health Research Center, St-Petersburg, Russia

**Keywords:** Chukotka, Yakutia, Russian Arctic, water supply, water accessibility, drinking water quality, water treatment, sanitation, sewerage, waste water

## Abstract

Information from 2013–2015 have been analysed on water accessibility, types of water service to households, use of water pretreatment, availability of sewerage, use of sewage treatment in Chukotka Autonomous Okrug and Yakutia Republic, based on evaluation information accessible in open sources, such as regional statistics and sanitary-epidemiologic reports. The main causes of the poor state of water supply and sanitation in the study regions include: very limited access to in-home running water (one-quarter of settlements in Chukotka and half of settlements in Yakutia have no regular water supply) and lack of centralised sewerage (78% and 94% of settlements correspondingly have no sewerage); lack of water pretreatment and sewage treatment, outdated technologies and systems; serious deterioration of facilities and networks, frequent accidents; secondary pollution of drinking water. Lack of open objective information on Russian Arctic water supply and sanitation in the materials of the regional and federal statistics hampers the assessment of the real state of affairs. The situation for water and sanitation supply in these Russian Arctic regions remains steadily unfavourable. A comprehensive intervention from national and regional governmental levels is urgently needed.

The United Nations Sustainable Development Goal (SDG) #6, aims to “achieve universal and equitable access to safe and affordable drinking water for all” by 2030 [1]. Most of the global population with unmet needs for water, sanitation and hygiene (WASH) are found in tropical or subtropical regions. However, it is not well understood that many Arctic residents lack WASH services and that the challenges of providing WASH differ in the Arctic and are incompletely understood. In addition, climate and environmental change is threatening existing water/sanitation systems by affecting water availability, making water and sewer treatment more complicated and disrupting distribution of water and sewage, particularly in areas with extensive permafrost thawing, flooding or erosion problems. This is more than a problem of inconvenience, inadequate access to WASH is known to have negative impacts on health in Arctic populations [2].

In 2015, an Arctic Council Sustainable Development Workgroup initiative was begun to document the extent of water and sanitation services in Arctic Nations, the related health indicators and climate-related vulnerabilities to WASH services [3]. As part of that effort, two international conferences were held in 2016 to highlight Arctic WASH problems and solutions. (ARTEK (Greenland) [4], WIHAH (Alaska) [5]). The Alaska meeting featured summary presentations on the status of WASH for each of the Arctic Nations where unmet WASH needs remain a problem (US, Canada, Greenland and Russia). This paper summarises the oral presentation prepared for the Alaska meeting by the author.

The poor state of water supply systems, shortage of water purification facilities and disinfection systems, and the generally low quality of drinking water in Russia and the Russian Arctic are publicly recognised and described in the Russian national literature. This includes a publication [6] where we focused on chemical and biological contamination of water sources and drinking water in 18 regions of the Russian Arctic, Siberia and Far East. A related publication assessed the levels of food-and-water borne diseases in those regions [7].

The present report was aimed at evaluating the water supply and sanitation in two Far Eastern regions of Russian Arctic – the Chukotka Autonomous Okrug and Yakutia (Sakha) Republic. This report will review information regarding water accessibility, types of service water to households, use of water pretreatment, availability of sewerage, and use of sewage treatment in these two regions. This report will highlight the public health challenges for WASH service in these regions.

To accomplish this a review was made information accessible in the open sources. The author’s personal experience in multiple scientific expeditions and long visits to Chukotka and Yakutia helped provide context to the available data. Few peer reviewed publications on water supply and sanitation in the Russian Arctic available and none exist for Chukotka and Yakutia. Official state and regional statistical materials are scanty, limited and not unified, which impede comparisons of the regions. However, data for 2013–2015 for this study were drawn from existing regional statistics, regional annual reports (titled: “Status of Sanitary-Epidemiological Wellbeing”), materials of regional committees and organizations and web resources. Searching and collecting web data was based on the key words: “water supply”, “sanitation”, “Chukotka” and “Yakutia”. Absolute values of some parameters of water supply and sanitation in the studied regions (if available) have been calculated into the relative values using demographic data from local statistics.

## Russian legal base in water supply and sanitation

The UN SDG objective for WASH for all people is also reflected in Russian law and policy. Russian Federal Law №52 (dated 1999) “On Sanitary and Epidemiological Welfare of the Population” [8] declares “The Right to Water” for the Russian population throughout the country: “Population of urban and rural areas should be provided with drinking water as a priority in an amount sufficient to meet the physiological and domestic needs”. This also contains the right to good quality drinking water: “Drinking water must be safe in epidemiologic and radiation context, harmless chemically and should have favourable organoleptic properties”, and indicates the responsibility of suppliers: “Organizations engaged in supplying of cold and hot water via centralized systems, are obliged to ensure the correspondence of drinking and hot water quality to the sanitary and epidemiological requirements”.

## Geography and demography

**Figure 1.**
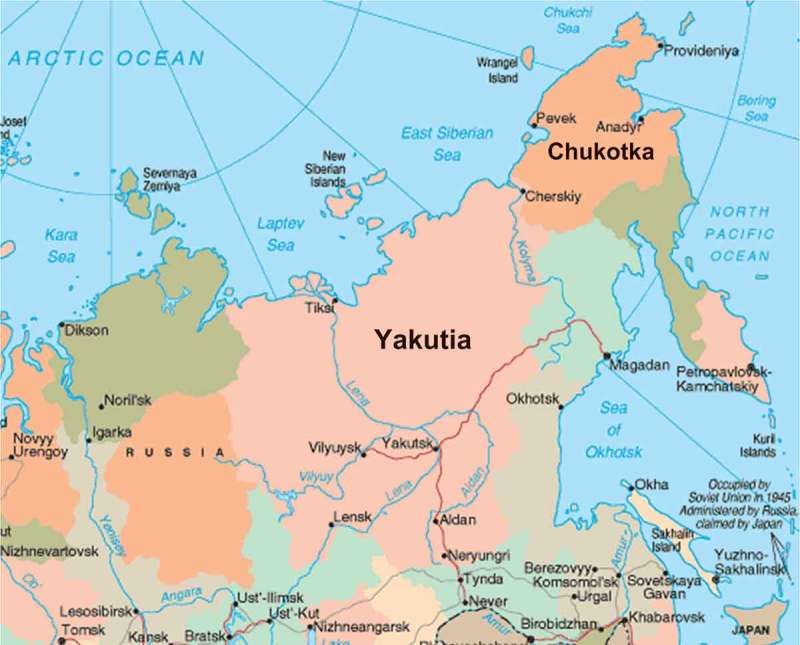
Map of eastern Russia (open access), showing the location of the Yakutia Republic and Chukotka Autonomous Okrug.

Table 1 provides the geographic and administrative-demographic characteristics of the studied regions. Yakutia Republic is four times larger and has a population 20 times greater than Chukotka Okrug (Figure 1), but the administrative territories are similar in terms of low population density. Chukotka’s 0.07 people/km^2^ is the lowest in Russia. Most settlements in Chukotka are scattered over vast distances. There are no railroads in Chukotka and no highways (asphalt roads). Transportation connections between settlements is possible in the summer only by air or water (with exception of few short dirt roads), or in winter – by temporary “winter roads” using off-road vehicles or powerful trucks. The transportation situation in northern Yakutia is similar to Chukotka. In southern Yakutia one short railroad (from Amur Oblast) and the Federal highway (through Yakutsk city to Magadan Oblast) are functioning all the year round.

**Table 1.** Demographic features of Chukotka Autonomous Okrug and Yakutia Republic, 2015 (adapted from [9,10]).

	Chukotka Autonomous Okrug	Yakutia Republic
Territory, km^2^	721,5 km^2^	3083,5 km^2^
Districts, number	6	36
Inhabited localities, number	44	641
Largest city, population	Anadyr – 14,900	Yakutsk – 303,800
Population total	50,160	959,700
Population density, people/km^2^	0.07	0.31
Urban population (% of the total)	34,720 (69%)	627,750 (65.4%)
Rural population (% of the total)	15,440 (31%)	331,950 (34.6%)
Indigenous population groups:		
people (% of the total)	Chukchi – 12,800 (25.3%)	Yakut – 467,000 (49.9%)
people (% of the total)	Eskimo (Inuit) – 1,500 (3%)	Evenk – 21,000 (2.2%)
people (% of the total)	Even – 1,400 (2.8%)	Even – 15,100 (1.6%)
people (% of the total)	Chuvants – 900 (1.8%)	
Non-indigenous population:		
Russians, people (% of the total)	25,100 (49.6%)	353,700 (37.8%)
Ukraine, people (% of the total)	2,900 (5.7%)	20,300 (2.2%)

## Water supply and sanitation

**Figure 2.**
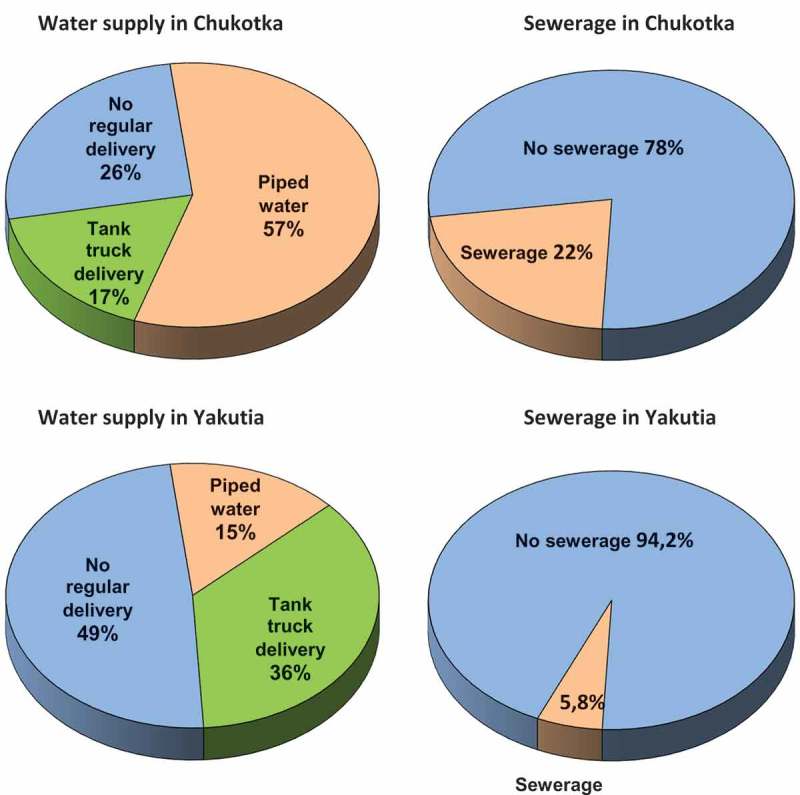
Water supply and sewerage in Chukotka and Yakutia, % of inhabited localities, 2014–2016 (created using [11,12,17]).

Figure 2 depicts the percentage of settlements (including all cities, townships and villages) where the water is provided either by centralised pipe (57% in Chukotka and 15% in Yakutia), or by tank truck delivery (17% and 36% correspondingly); one-quarter of settlements in Chukotka and half of the settlements in Yakutia have no regular water supply. Centralised sewerage is available in 22% of Chukotka settlements and only in 5.8% of Yakutian inhabited localities.

Tables 2–4 describe more detailed information on Yakutia; similar data on Chukotka are not accessible anywhere in the open sources. Centralised pipe water supply in Yakutia (Table 2) is available mainly in the cities (92%) and townships (86%) while only a few villages have running water (8.2%). This is similar for sewerage availability – less than 1% of villages have piped sewers.

**Table 2.** Number and share of inhabited localities with water pipe and sewerage in Yakutia (adapted from [12]).

Inhabited localities^a^	Total number in Yakutia	Number with piped water	% of water piped	Number with piped sewerage	% with sewerage
Cities	13	12	92.3	8	61.5
Townships	42	36	85.7	24	57.1
Villages	586	48	8.2	5	0.9
**Totally**	**641**	**96**	**15**	**37**	**5.8**

^a^Cities are larger than townships are larger than villages (determination is made at the regional level).

**Table 3.** Characteristics of housing water supply and sanitation in Yakutia, 2015 (adapted from [13]).

% of housing	Yakutia republic (%)	Yakutsk city (%)
In-home running water	53.3	79.7
In-home sewerage	52.3	76
In-home hot running water	50.6	72.6

**Table 4.** Share of housing in the cities with centralised in-home sewerage, Yakutia – 2013 (from [14]).

Cities	Total population number, 1,000 people	% of houses with in-home sewerage
Yakutsk	303.8	76.0%
Neryungri	57.8	35.4%
Mirnyi	34.8	68.7%
Lensk	23.7	31.0%
Aldan	20.9	34.2%
Vilyuisk	10.7	0
Nyurba	9.9	0
Olekminsk	9.2	0
Pokrovsk	9.1	26.0%
Srednekolymsk	3.5	0

Table 3 shows the proportion of housing units equipped with in-home piped water and sewerage in Yakutia. Yakutsk city with population of 304,000 people has a high percentage of in-home water (78%) and sewerage (76%). Yakutsk city (and several relatively large cities and townships) represent a large proportion of Yakutia’s population and are included in the total Yakutia Republic figures, and thus influence the total population statistics.

Table 4 shows the percentage of houses in Yakutian cities (largest inhabited locations) connected to the centralised sewerage – only Yakutsk city and Mirnyi city (the diamond “capital” of Russia) have greater than 50% of houses with in-home sewerage (76% and 69% correspondingly) while in other cities this index do not exceed 35%. In the townships, with a population of 3,000–11,000 people, houses do not have sewerage service, with the exception of Pokrovsk township.

Therefore, using the Yakutian water supply and sanitation data we see that in an officially “served” inhabited location (whether it is urban or rural) the percentage of “served” houses is not high. Documentation of WASH service for homes in Chukotka is harder to establish, due to a lack of open-source or published data. For example, the following quote from a State Report [15] declares high levels of water service: “Centralized running water supply in Chukotka Okrug is carried out in 25 inhabited locations with population of 47,055 people, or 92.6% of total Chukotka Okrug population”. However, specific household or village-level data are not available to support this statement.

This shows the difficulty of identifying the proportion of houses and the overall population who have WASH service in Yakutia and Chukotka using the available official statistics. Therefore, to gain an accurate picture of the situation further investigation of each inhabited location is needed to compile data on houses, families and persons who have WASH service.

Water treatment facilities in Chukotka are available only in four cities (Anadyr, Bilibino, Pevek and Provideniya); elsewhere in Chukotka only raw, untreated water is available. Most aqueducts in small Chukotka settlements begin from the nearest lakes; none of them are equipped with water treatment systems [16]. In small coastal villages in Chukotka (e.g. those on the Chukotka Sea and Bering Sea), water is typically delivered through trucked service. This water is often obtained from local lakes and rivers, and is not treated before delivery to households. The water is typically unloaded into large barrels (200–250 l) within the home by means of a hose, which is fed through a nearby window. These water barrels generally have no attached pipes or drains, and water is removed with a ladle by dipping it into the surface of the water. For hand-washing and tableware washing the people use a wall hanging water dispenser (called in Russia “rukomojnik” filled manually by ladle) over the sink with a hole; the waste water drains into a bucket which is typically emptied outdoors on the ground.

Specific information about the use of raw untreated water delivered by tank trucks in Yakutia is unavailable, but based on personal experience use of raw water appears to be common in small settlements in this region. For example, during winter in Yakutia, household water is often provided only by ice blocks from untreated sources. In some settlements this is a common private business and people who are not able to collect ice for themselves (the old, sick or handicapped) pay the ice-sellers for their water needs.

## Conditions of facilities, equipment and technologies

In Yakutia the majority of water intake facilities and water treatment systems were installed in 1960’s and 1970s. Since that time no improvements of technologies and equipment, no overhaul of facilities or networks, no replacement of old ones have been carried out [16] except for minor repairs. The percentage of the centralised water supply and sanitation systems which need to be replaced is 75% averagely in Yakutia and 65% in Yakutsk city [13]. Yearly spring floods negatively affect the quality of surface water sources in Yakutia which significantly worsens the quality of drinking water. Existing water pretreatment facilities do not provide adequate water quality due to low efficiency (deterioration, old technologies). In Yakutsk city the water pretreatment is not carried out (no flocculation or filtration), water disinfection is conducted only by addition of liquid chlorine [16].

In Chukotka the poor quality of centralised drinking water is due to many factors, including “the lack of water pre-treatment and water disinfection at all water supply facilities” (except in four cities), “use of obsolete technologies, lack of sanitary protection zones of water bodies, pollution of the catchment areas and adjacent shore areas” [17]. Also, it is common for “secondary contamination of water to occur because of deterioration and corrosion of pipelines and distribution networks (40–80% of water supply systems in Chukotka settlements need to be replaced)”, “the formation of dead-end lines of water pipes (rather than circular) which causes stagnation of water in the networks and increases the pipeline corrosion processes” [17]. For example, “in Anadyr city there are 16 dead ends. Further, a lack of sufficient serviceable butterfly valves hampers the isolation of pipelines sections for their regular cleaning, disinfection and repairing” [17].

## Sewerage service

Centralised sewerage in Chukotka exists only in 10 towns (22% of settlements). Sewage treatment facilities are absent in all Chukotka – untreated sewage waters (total volume > 5 million cubic meters per year) go directly to rivers, lakes and then to the sea. For example, in Anadyr City the untreated sewage (an estimated 1.8 million cubic meters per year) is released into the Kazachka river which flows into to Anadyr Bay and to the Bering Sea [17]. In the author’s experience, sewage drains in some townships the drains go to cesspools and after pumping out by a tank-truck, are delivered and discharged onto the ground surface or nearest body of water. The use outdoor toilets and self-haul systems are common, including the indoor use of bucket with subsequent empting near the habitation. Plastic bags to contain waste (“honey-buckets”) are not used and there are no special service for collecting and disposing of waste in communities. Very rarely has the author seen modern private cottages where the owners have their own toilets with septic systems. Quite often within one settlement many types of toilet systems are in use.

## Challenges and governmental “clean water” plans

In December 2010 the Federal Target programme “Clean water” for the period 2011–2017 was adopted by the Russian Government [18]. The main goal of the programme was to provide the population with sufficient quantity of safe and harmless drinking water via modernization and development of water supply and sanitation sector. Regional target subprograms started in many Russian regions, including Arctic Yakutia and Chukotka. However, the collapse in oil prices and the double fall of the Ruble rate in 2014 created a subsequent economic crisis, which led to the failure of various Federal programmes. According to the Russian Accounting Chamber, the implementation of “Clean water” programme was suspended in 2014 due to “inefficient program management” [19]. Lack of finances appears to be an ongoing for the realisation of the programme in the Arctic. The Arctic regions of Russia are financially dependent on the Federal center, despite their rich mineral production. Chukotka and Yakutia produce gold (each averaging 20–30 tons per year) [20,21], and Yakutia is one the world biggest producer of diamonds [22]. However finances are not the only aspect of the water problem in these regions. The crisis of water supply and the alarming water security situation in the Russian Arctic is a complex issue of legal, administrative, financial, economic, technologic, ecologic and other problems. It is clear that the actual status of the problem in Yakutia and Chukotka demands an urgent and comprehensive intervention. However, even though there is a strategy specific to these regions, it is unclear when this will improve the situation.

## Conclusions

The water supply and sanitation situation in the Russian Arctic Yakutia and Chukotka is remarkable for the very limited access to running in-home service. Additionally, the combination of a lack of water pretreatment, common use of raw surface water, and direct sewage discharge into the environment all pose real and ongoing threats to public health. Many of the systems are old and subject to deterioration. The history of waterborne disease outbreaks in the region further indicate that public health has suffered from this situation. The difficulty in obtaining information on Russian Arctic water supply and sanitation in the materials of the regional and federal statistics hampers the assessment of WASH status for much of the population and limits ongoing evaluations of progress. Therefore, for these regions it will continue to be difficult to report any progress towards SDG#6 without better collection and release of data and a renewed effort to accomplish the goals of the 2010 Clean Water decree.
